# Sequential measurement of the neurosensory retina in hypertensive disorders of pregnancy: a model of microvascular injury in hypertensive emergency

**DOI:** 10.1038/s41371-021-00617-1

**Published:** 2021-10-08

**Authors:** Robert J. Herman, Anshula Ambasta, R. Geoff Williams, Kelly B. Zarnke, Fiona E. Costello, Mingkai Peng, T. Lee-Ann Hawkins

**Affiliations:** 1 Division of General Internal Medicine, Department of Medicine, Cumming School of Medicine, University of Calgary, Calgary, AB Canada; 2 Libin Cardiovascular Institute, Cumming School of Medicine, University of Calgary, Calgary, AB Canada; 3 O’Brien Institute of Public Health, Cumming School of Medicine, University of Calgary, Calgary, AB Canada; 4 Alberta Health Services, Calgary Zone, Calgary, AB Canada; 5 Section of Ophthalmology, Department of Surgery, Cumming School of Medicine, University of Calgary, Calgary, AB Canada; 6 Department of Clinical Neurosciences, Cumming School of Medicine, University of Calgary, Calgary, AB Canada; 7 Obstetric Internal Medicine, Department of Medicine, Cumming School of Medicine, University of Calgary, Calgary, AB Canada

**Keywords:** Pre-eclampsia, Blood flow

## Abstract

Optical coherence tomography of the eye suggests the retina thins in normal pregnancy. Our objectives were to confirm and extend these observations to women with hypertensive disorders of pregnancy (HDP). Maternal demographics, clinical/laboratory findings and measurements of macular thickness were repeatedly collected at gestational ages <20 weeks, 20-weeks to delivery, at delivery and postpartum. The primary outcome was the change in macular thickness from non-pregnant dimensions in women with incident HDP compared to non-hypertensive pregnant controls. Secondary outcomes were the relationship(s) between mean arterial pressure (MAP) and macular response. Data show macular thicknesses diminished at <20 weeks gestation in each of 27 pregnancies ending in HDP (mean 3.94 µm; 95% CI 4.66, 3.21) and 11 controls (mean 3.92 µm; 5.05, 2.79; *P* < 0.001 versus non-pregnant dimensions in both; *P* = 0.983 HDP versus controls). This thinning response continued to delivery in all controls and in 7 women with HDP superimposed on chronic hypertension. Macular thinning was lost after 20 weeks gestation in the other 20 women with HDP. MAP at loss of macular thinning in women without prior hypertension (*n* = 12) was identical to MAP at enrollment. However, mean MAP subsequently rose 19 mmHg (15, 22) leading to de novo HDP in all 12 women. Loss of thinning leading to a rise in MAP was also observed in 8 of 15 women with HDP superimposed on chronic hypertension. We conclude the macula thins in most women in early pregnancy. Those who lose this early macular thinning response often develop blood pressure elevations leading to HDP.

## Introduction

Hypertensive disorders of pregnancy (HDP), including preeclampsia (PE), gestational hypertension (GHTN) and pre-existing chronic hypertension (cHTN) are common. In aggregate, they occur in 7–10% of all pregnancies and worldwide are a leading cause of maternal and perinatal morbidity and mortality [1]. The pathogenesis of new onset hypertension or worsening of pre-existing hypertension in pregnancy in otherwise young, healthy women is not known.

Early imaging studies looking at the brain in patients with non-pregnant forms of severe hypertension point to breach of the upper threshold of autoregulation with hyperperfusion and vasogenic edema as the cause of microvascular CNS injury in hypertensive emergency [2]. Clinically, this is referred to as posterior reversible encephalopathy syndrome (PRES). All women with eclampsia [3, 4] and at least 10–20% of women with PE [5, 6] possess these radiologic abnormalities. However, 20–40% of patients with PRES, including many women with HDP have low or an insufficient elevation in systemic blood pressure to “run” expected autoregulatory thresholds in the microcirculation of the brain [2]. This has led some to suggest vasoconstriction/ischemia is the cause of microvascular injury [7].

The neurosensory retina is unique in that it possesses 2 blood retinal barriers; one at its capillary interface with the central retinal artery regulating movement of water into this tissue, the other at the retinal pigment epithelium (RPE) where cells that comprise this barrier pump chloride ion and lactate to its outward-facing side (see Supplementary Data Fig. S1). The purpose of this is to generate a gradient capable of drawing water across large, avascular areas of the eye [8] and then shuttling it to the choroid circulation where it exits the eye. Thus, the neurosensory retina has a finite dimension dependent upon both autoregulation and this gradient at the outer blood retinal barrier. Spectral domain optical coherence tomography (SD-OCT) has a spatial resolution 3 orders-of-magnitude greater than CT or MRI and is able to accurately measure the thickness of the neurosensory retina. We hypothesized that women with HDP whose circulations approach and in some instances breach the limits of microcirculatory control may show evidence of vasoconstriction/hypoxia or edema within this tissue. Our objectives, therefore, were to prospectively measure macular thicknesses in non-hypertensive pregnant controls and in women who go on to develop HDP in order to determine when and how this injury occurs.

## Subjects and methods

### Study populations, inclusion and exclusion criteria

Pregnant women aged 18–45 years and at gestational age <20 weeks were recruited from an outpatient obstetric internal medicine clinic into one of two risk categories relating to their likelihood of developing of HDP. “Low-risk” women possessed no high-risk criteria for the development of HDP. “High-risk” women were defined by the presence of cHTN, clinical high-risk predictors for HDP including prior PE, GHTN, or chronic kidney disease [9], or ≥2 of the following established risk factors: age >35 years, body mass index >30 kg/m^2^, multiple gestation pregnancy and non-Caucasian ethnicity [10]. Women with pre-existing diabetes mellitus, vasculitis, or retinal disease were excluded from the study. Participants were then followed through pregnancy and into the postpartum period where final allocation to the non-hypertensive control or HDP cohorts was made. Women who developed gestational diabetes or delivered babies with fetal anomalies were removed from these cohorts. Only participants having complete data (i.e., at least 1 study-related encounter in each of the 3 gestational intervals plus a non-pregnant encounter) were included in the final analyses.

### Study design and methodology

The study was designed as a prospective observational cohort study with repeated measures at <20 weeks gestation, between 20 weeks and delivery, at delivery and in the non-pregnant state. Non-pregnant imaging was acquired postpartum; >2 months from delivery in those without evidence of microvascular injury and >6 months if microvascular injury was considered. This timing was to align with data on recovery of vasogenic edema from the brain in PRES [4, 11]. Recruitment began in October 2013 and clinical follow-ups were completed in May 2018. Data collected at enrollment and at each clinical encounter included participant age, gestational age, medical co-morbidities, medications, clinical symptoms, height, weight, office blood pressure and the results of all investigations ordered by physicians. Blood pressure was measured using an automated oscillometric device with the patient seated and arm supported at the level of the heart following 5-minutes rest. A large adult cuff was used if mid-arm circumference measured ≥34 cm. Funduscopic examination of both eyes was performed by a physician trained in identifying hypertensive retinopathy and included visualization of the optic nerve head, superior and inferior nasal and temporal arcades and the fovea. Standard research definitions were used to diagnosis HDP and to classify participants by subtype and severity [12, 13]. Obstetrical and neonatal outcomes were collected from standardized birth and delivery records. The Conjoint Health Research Ethics Board at the University of Calgary approved our study (CREB 15-0374, January 17, 2013). All participants provided written informed consent. This study was registered with the US National Library of Medicine (NCT04286217).

### SD-OCT measurement

Eye measurements were obtained using a standardized approach and the same Zeiss Cirrus 4000 SD-OCT instrument. Two SD-OCT retinal images were taken from each eye at every encounter without dilation of the pupil at a scanning density of 512 A-scans x 128 B-scans over a 6 mm square grid focused on the fovea. Between sequential images (i.e., right eye first measurement, right eye second measurement, etc) participants were instructed to remove their face from the examining platform and the instrument reset to default in order to match the effect of an independent scan. Scans were examined systematically for signal strength, definition of vessel architecture, centrality and motion artifact to determine image quality, and the better of the 2 was prospectively selected for analysis. Segmentation was verified on every scan. Macular thickness is a standard measure calculated by the instrument from the internal limiting membrane to the retinal pigment epithelium. Scans were referenced to a control image obtained in the same individual in the non-pregnant state. A certified ophthalmologist reviewed all SD-OCT images.

### Outcomes

The primary outcome was the change in macular thickness from its non-pregnant baseline over the 3 gestational intervals in women with HDP compared to non-hypertensive pregnant controls. Secondary outcomes were the differences in macular response between subgroups with incident HDP (i.e., de novo HDP, HDP superimposed on cHTN, PE, GHTN) and their relationships to systemic blood pressure.

### Statistical analyses

Preliminary data revealed an effect size of ≥4 µm change in macular thickness and a probability of developing PE of 50% in high-risk cohorts. The probability of correctly detecting retinal injury in those with PE was 63%, and 33% in those (1 - PPV) without that outcome, giving a sample size estimate of 20 controls and 40 HDP. These sample sizes were increased to 30 and 125, respectively, to allow for dropouts, missing data and inclusion of a planned secondary outcome of those with and without cHTN.

Given the exploratory nature of our study and its small subject numbers, we considered both summary statistics and measures of individual response. In respect to the latter, a decision support tool was derived whereby clinically meaningful change was arbitrarily defined as a directionally identical difference ≥ ±4 µm (two times the test re-test coefficient of repeatability of our instrument) in 3 or more contiguous segments on the Early Treatment of Diabetic Retinopathy Study [14] (ETDRS) grid in a single eye. This summary measure was tested against the frequency of appearance of all combinations of lesser levels of change in a null difference distribution comprised of 1530 paired images collected at the same encounter. It identifies an interval change greater than the expected null difference at a *P* = 0.047. The decision support tool was used to allocate participants into HDP sub-cohorts according to their macular response. This formed the basis of our stratified analysis. The 2 eyes were considered independent variables in summary analyses (it is not uncommon to see unilateral PRES or papilledema). However, both eyes had to be normal in order to classify an individual as having no evidence of microvascular injury.

Participant characteristics were summarized using means and standard deviation (SD) for continuous variables and frequencies for categorical variables. A linear, mixed effects model was used to estimate macular thickness in the 3 gestational intervals with random effects considered at three hierarchical levels: patient level, eye side (right or left) and position on the ETDRS grid. A continuous autocorrelation structure was applied to adjust for correlation of variables repeatedly measured at differing clinical encounters. Ninety-five per cent confidence intervals were computed for all outcome variables. Multiple regression analysis was performed to verify the findings of the stratified analysis and to identify the effects of potentially confounding variables. Statistical significance was a *P* < 0.05.

## Results

### Main participant characteristics

**Fig. 1:**
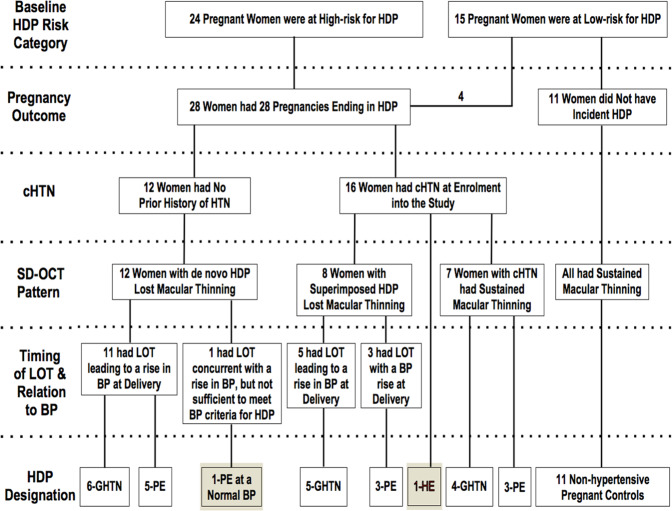
Clinical pathways and outcomes of 11 non-hypertensive pregnancies and 28 pregnancies ending in HDP. Pathways move vertically across horizontal fields identified along the left margin of the Figure from baseline risk for HDP at enrollment into the study to HDP sub-type designation (preeclampsia, gestational hypertension or hypertensive emergency) at delivery. Abbreviations: HDP hypertensive disorder(s) of pregnancy, cHTN chronic hypertension diagnosed or on treatment at enrollment or persistent elevations in blood pressure before 20 weeks gestation, HTN hypertension, SD-OCT spectral domain optical coherence tomography, LOT loss of macular thinning, BP systemic blood pressure, GHTN gestational hypertension, PE preeclampsia, HE hypertensive emergency. Technical Format: black and white.

Maternal co-morbidities and demographics of the 11 control and 28 incident HDP participants appear in Table 1. Overall, 8 women (29%) were of non-Caucasian descent and 16 women (57%) had cHTN. The clinical course of these individuals from inception to outcome at delivery is plotted in Fig. 1. Overall, the HDP cohort was slightly older with a mean (SD) age of 33.4 (3.8) years (*P* = 0.51) and mean weight 11.3 kg heavier at 76.2 kg (15.8; *P* < 0.05) compared to non-hypertensive controls. Mean gestational age at enrollment was similar (*P* = 0.81) in the 2 cohorts. However, gestational age at delivery was 37 weeks 2 days (*P* < 0.05), almost 2 weeks earlier due to an expedited delivery in 27 of 28 HDP pregnancies. Delivery was by caesarean section in 16 women (57%). Twenty-seven HDP pregnancies ended in a singleton live birth; 1 woman delivered twins. There were no fetal losses. The incidence of postpartum complications was higher in women with HDP (54%) and was commonly associated with postpartum hemorrhage. Three of 28 pregnancies had fetal growth restriction (11%) as defined by standard criteria [15]. The timing of the non-pregnant encounter averaged 31 weeks (95% CI 20, 42; *P* = 0.25) in both cohorts. Fundoscopy revealed diffuse arteriolar narrowing in 4 cases. One participant had clinical and OCT evidence of new bilateral papilledema. There were no SD-OCT findings of sub-retinal edema or detachment, retinal vessel occlusion, or neovascularization.

**Table 1:** Maternal co-morbidities in controls and HDP by subtype.

Age	Co-morbidities	nC	HRx	pH	PE	sPE	KD	CTD	Ob
33	Chronic DVT/new clot on sAC								
33	Hashimoto’s thyroiditis - on T4 replacement								
19	Migraine headaches								
36	Sagittal vein thrombosis - resolved on sAC								
39	AT-III deficiency - on sAC								
34	Family history of VTE								
38	Sickle cell anemia on exchange transfusion	x							
27	Graves disease in remission	x							
34	OCP-induced hypertension - resolved								
30	Prior HELLP syndrome				x	x			
33	ADHD on dexidrine								
33	No co-morbidities	x							
31	AT-III deficiency/new DVT on sAC								
33	Protein S deficiency/hypothyroid on T4				x				
32	Homozygous factor V leiden/SVT				x	x			
39	Hypothyroid on T4	x			x	x			x
31	Non-alcoholic fatty liver disease				x				x
33	Chronic neutropenia								
30	May thurner syndrome/factor V leiden/VTE								x
39	Premature ventricular contractions								
36	SLE with kidney involvement/APLS	x					x	x	
28	Prior HELLP with DIC and hepatic bleeds				x	x			
31	IgA nephropathy with FSGS						x		
32	cHTN/ADPKD		x		x	x			
29	cHTN	x	x						
44	SLE in remission/cHTN/hypothyroid on T4		x		x			x	x
27	cHTN/obstructive sleep apnea	x		x					x
36	cHTN	x	x		x				x
35	cHTN								x
30	Membranoproliferative GN/cHTN		x		x		x		x
34	cHTN				x	x			x
36	cHTN			x					x
35	ADPKD/cHTN				x	x	x		
33	PAN/nephrotic syndrome/cHTN/Crohn’s		x				x	x	
33	Crohn’s/collapsing FSGS/cHTN		x	x			x	x	
39	Crohn’s/celiac/cHTN/graves disease		x		x	x		x	
28	New diagnosis of severe cHTN		x	x					
33	Raynauds/prinzmetal angina								
35	Hypertensive emergency		x						x
Percentage of the total		29	36	14	41	58	21	18	39

The upper panel represents non-hypertensive pregnant controls. The second panel includes participants with de novo HDP. The third panel includes participants with HDP superimposed on chronic hypertension where loss of thinning occurred. The fourth panel includes participants with chronic hypertension who maintained macular thinning to delivery. The final panel is a patient who had a hypertensive emergency at the time of enrollment.

*HDP* hypertensive disorder(s) of pregnancy, *nC* non-Caucasian ethnicity, *HRx* hypertension on treatment with medication at enrollment, *pH* poorly controlled hypertension at enrollment, *PE* prior history of preeclampsia or other subtype of HDP, *sPE* prior severe preeclampsia, *KD* chronic kidney disease, *CTD* chronic inflammatory conditions such as crohn’s or a connective tissue disease, *Ob* BMI > 30 kg/m^2^, *DVT* deep vein thrombosis, *sAC* systemic anticoagulation, *T4* thyroxin, *AT-III* antithrombin III, *VTE* venous thromboembolism, *OCP* oral contraceptive pill, *HELLP* hypertension elevated liver enzymes low platelets proteinuria, *ADHD* attention deficit hyperactivity disorder, *SVT* superficial vein thrombosis, *SLE* systemic lupus erythematosis, *APLS* anti-phospholipid antibody syndrome, *DIC* disseminated intravascular coagulopathy, *IgA* immunoglobulin A, *FSGS* focal segmental glomerular sclerosis, *cHTN* chronic hypertension, *ADPKD* autosomal dominant polycystic kidney disease, *GN* glomerulonephritis, *PAN* polyarteritis nodosa, *Crohn’s* crohn’s disease, *Celiac* celiac disease, *Graves Disease* graves disease without eye involvement.

### SD-OCT findings in women with hypertensive disorders of pregnancy

One participant had a hypertensive emergency with evidence of retinal injury at enrollment. This was not known until completion of her final postpartum scan. She was removed from all subsequent analyses, but her case brings important information to bear and will be discussed separately. Of the remaining 27 HDP participants, 26 demonstrated macular thinning in the first 20 weeks of gestation and all 27 had thinned by 32 weeks. The pattern and extent of thinning (bilateral, confluent 4–6 µm loss in thickness, greatest in the inner 1–3 mm circle of the ETDRS grid) was identical to that of non-hypertensive pregnant controls (Table 2). Whereas macular thinning was maintained and greatest at the time of delivery in controls, 20 of 27 women with HDP (74%) went on to lose part or all of this early pregnancy thinning response at or before delivery. This was seen as early as 16 weeks gestation, but more commonly occurred after 25 weeks.

**Table 2:** Estimated change in macular thickness* (µm) in women with HDP vs controls at < 20 wks gestation.

Type of pregnancy	Mean (95% CI)	*P* value vs controls
HDP (*n* = 27)	−3.94 (−4.66, −3.21)	0.983
Controls (*n* = 11)	−3.92 (−5.05, −2.79)	

*Compared to baseline thickness in the non-pregnant state.

*µm* micrometers, *HDP* hypertensive disorder(s) of pregnancy, *vs* versus, *wks* weeks, *CI* confidence interval, *P value* probability threshold to indicate statistical significance.

Loss of macular thinning was generally patchy, partially effacing, and progressive. Only the participant excluded for severe hypertensive emergency had overt thickening of her maculae (Fig. S2 Case 2 Supplementary Data). Loss of thinning was limited to a single eye in 7 participants (35%). Finally, macular thinning was maintained throughout the entire gestational period in 7 of the 27 women with HDP (26%; 16 of 16 eyes), indistinguishable from that of non-hypertensive controls. Estimated changes in retinal thicknesses over the 3 gestational intervals by retinal response pattern and the presence or absence of pre-existing hypertension are shown in Table 3. Absolute macular thicknesses for the same data (Tables S1, S2) and the results of the regression analyses (Table S3) can be found in Supplementary Data titled after those topics. Fractional thinning of vascularized and non-vascularized tissues, including the internal and external retina and choroid and their meaning appear in the Fractional Thinning Section of the Supplementary Data.

**Table 3:** Estimated change* in macular thickness (µm) from < 20 wks to delivery in controls and HDP by HDP subtype.

Type of pregnancy	At < 20 wks mean (95% CI)	At 20–40 wks^†^ mean (95% CI)	*P* value vs < 20 wks	At delivery mean (95% CI)	*P* value vs < 20 wks	*P* value vs 20–40 wks
De novo HDP (*n* = 12)	−4.35 (−5.48, −3.22)	−2.99 (−4.10, −1.88)	<0.001	−3.45 (−4.58, −2.32)	<0.001	0.014
HDP/cHTN without LOT (*n* = 7)	−3.91 (−5.14, −2.59)	−3.80 (−5.02, −2.59)	0.481	−4.46 (−5.70, −3.23)	<0.001	<0.001
HDP/cHTN with LOT (*n* = 8)	−3.39 (−4.37, −2.40)	−2.85 (−3.83, −1.86)	<0.001	−1.06 (−2.08, −0.04)	<0.001	<0.001
Non-hypertensive Controls (*n* = 11)	−3.92 (−5.07, −2.77)	−3.08 (−4.33, −1.83)	<0.001	−5.16 (−6.41, −3.91)	<0.001	<0.001

*Compared to baseline thickness in the non-pregnant state.

^†^20–40 weeks means from 20 weeks gestation up to, but not including delivery. Delivery was before 40 weeks in all but 1 woman with HDP.

*µm* micrometers, *wks* weeks gestation, *HDP* hypertensive disorder(s) of pregnancy, *CI* confidence interval, *P* *value* probability threshold to indicate statistical significance, *vs* versus, *cHTN* chronic hypertension, *HDP/cHTN* HDP superimposed on cHTN, *LOT* loss of thinning.

### Relationship of blood pressures to SD-OCT findings in de novo HDP

Twelve of the 20 pregnancies (60%) where HDP was associated with loss of macular thinning occurred in women with no prior history of hypertension. Average MAP at enrollment in these 12 individuals was 84 mmHg (79, 89), or 112/70 mmHg by conventional methods of reporting. This was not meaningfully different from MAP in non-hypertensive pregnant controls (*P* = 0.96). MAP at the time of loss of thinning was 87 mmHg (82, 92), similar to MAP at enrollment (*P* = 0.19). However, in the 2–8 weeks that followed MAP rose on average 19 mmHg (15, 22) to a mean MAP of 101 mmHg (94, 107) at delivery (*P* < 0.001 compared to both earlier measurements). The only participant not showing this pattern of loss of thinning at a normal systemic blood pressure followed by a meaningful rise in MAP presented at 37 weeks gestation with a systemic blood pressure of 123/79 mmHg (MAP up 8.3 mmHg from previous measurements), new onset proteinuria and severe fetal growth restriction/fetal distress leading to urgent caesarean section. Her clinical diagnosis was PE at a normal blood pressure. The diagnosis in the other 11 participants was PE in 5 women (42%), of which 2 (40%) were severe, and GHTN in 6 women.

### Relationship of blood pressures to SD-OCT findings in HDP superimposed on cHTN

Eight of 20 HDP pregnancies (40%) with loss of macular thinning occurred in women with HDP superimposed on cHTN (*n* = 15). Six were on anti-hypertensive treatment at enrollment; all were on treatment by delivery. Three participants had an accelerated clinical course where systemic blood pressures rose steeply with loss of thinning first noted at delivery. Thus, the true MAP at loss of thinning could not be reliably estimated in these individuals. All 3 were classified as PE, 2 of which were severe. In the other 5, mean treated MAP at loss of thinning was 98 mmHg (91, 105), 11 mmHg higher than MAP at enrollment (*P* < 0.05) and 11 mmHg higher than MAP at loss of thinning in de novo HDP (*P* < 0.03). As in de novo HDP, loss of thinning was followed by an 8–20 mmHg (median 14) rise in systemic blood pressure to a mean treated MAP at delivery of 102 mmHg (97, 108). All 5 were classified as GHTN.

Macular thicknesses remained consistently thinned from <20 weeks to the time of delivery in 7 of 15 pregnancies (47%) in women with HDP superimposed on cHTN. Four were on anti-hypertensive treatment at enrollment; all but one was on treatment by delivery. MAP rose sharply in late pregnancy by 23–28 mmHg in all 7 women to a mean maximal MAP of 117 mmHg (105, 129), or 150/100 mmHg. These were the highest mean MAP of any cohort in our study. Two of the 7 pregnancies were classified as PE. Both were early onset and severe and both had intrauterine growth restriction. The other 5 were classified as GHTN.

### SD-OCT findings of hypoperfusion injury in HDP

Five women with HDP (17%) developed sudden and confluent macular thinning concomitant with a significant drop is systemic blood pressure most often due to peripartum hemorrhage. Antecedent loss of thinning was present in 4 of the 5 cases. Two had de novo HDP; the other 3 had HDP superimposed on cHTN. Two of these participants are presented in Fig. S2 (Supplementary Data) to further characterize this important finding.

## Discussion

Our data supports earlier observations that the neurosensory retina thins in normal human pregnancy [16]. In contrast to that study which reports thinning of the subfoveal segment but no other region of the ETDRS grid and a subsequent study by the same group suggesting no differences at all [17], we show that thinning occurs broadly and confluently across the entire macula in every eye in every individual in early pregnancy. Unlike normal pregnancy where macular thinning is preserved and most robust at the time of delivery, this thinning response is lost in many women (74%) with incident HDP, including 9 of 12 women (75%) with preeclampsia. Three studies have used OCT to examine the macula in PE. Two were cross-sectional cohort studies; the first enrolled 27 women with late PE versus 25 healthy pregnant controls [16], the second enrolled 55 women with PE (severity not reported) and 43 healthy pregnant controls at 36-weeks gestation [18]. The third used older technology to study 22 women with early PE at 30 weeks and 24 healthy pregnant controls before and 3 days following delivery [19]. All 3 studies reported no differences between macular thicknesses in women with PE versus those with normal pregnancy. Our data also concur with these findings since initial thinning is fully effaced in most women with PE by the time of delivery. Only rarely did we observe overt thickening of the macula. None of these studies looked at early gestation, thus it is likely early macular thinning progressing to loss of thinning was simply missed. Others and we have looked for changes at the optic nerve head and peripapillary retinal nerve fiber layer in pregnancy. This is discussed in Supplementary Data under the heading “Are Changes … Found Elsewhere?”

Cardiac output increases 1.5-fold between 16 and 20 weeks gestation; circulating blood volume increases 1.3 to 1.4-fold by late pregnancy [20, 21]. These are large changes persisting over several months and are certain to challenge the microcirculation of many tissues. Flow-mediated vasoconstriction is a unique property of cerebral arteries [22, 23]; most other regional arteries dilate in response to increases in blood flow. The central retinal artery of the eye is known to autoregulate (multiple references, summarized by Kur et al. [24]), and vasoconstriction of that vessel would be expected to reduce net transudation into the neurosensory retina. While the extent of macular thinning observed in our study is not large, the fact the retina thins at all, is noteworthy. Indeed, the Starling equation for the convective movement of water into neural tissues predicts increases in blood flow and circulating blood volume, if unopposed, could lead to over-filtration and flooding of end-tissues, including the eye [25]. Thus, macular thinning in pregnancy signals the presence of a mitigating vasoconstrictive response. OCT angiography has recently localized that response to the central retinal artery where vascular perfusion density is reduced in the deep capillary plexus and parts of the superficial capillary plexus in women in late pregnancy with and without PE [18]. OCT angiography of non-pregnant men and women with very high systemic blood pressures also shows reduced perfusion in the deep plexus compared to age-matched non-hypertensive controls [26]. We propose gain-of-function of autoregulation by the central retinal artery is responsible for early pregnancy macular thinning in our study.

**Fig. 2:**
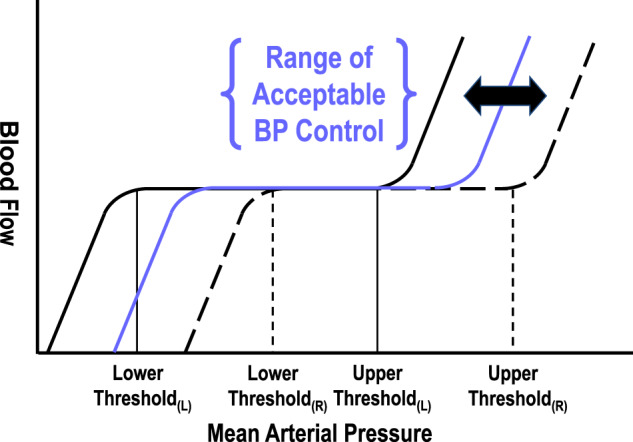
Effects of left-shifting or right-shifting of autoregulation on tissue tolerance to hypoperfusion or hyperperfusion injury. Lower and Upper thresholds of effective autoregulation for a left-shifted (solid lines) or a right-shifted (dashed lines) autoregulatory curve. A hypothetical normal autoregulatory curve and its upper and lower boundaries appear in blue. Range of Acceptable BP Control represents the theoretical range of systemic blood pressures considered safe or desirable within a population, as set by guideline. Note, leftward shifting protects the microcirculation at lower systemic blood pressures, but exposes tissues to hyperperfusion injury at systemic blood pressures within the upper ranges of acceptable BP control. Rightward shifting of autoregulation protects the microcirculation at higher systemic blood pressures, but exposes tissues to hypoperfusion injury at systemic blood pressures within the lower ranges of acceptable BP control. Abbreviations: BP systemic blood pressure, (L) Left-shifted autoregulation, (R) Right-shifted autoregulation. Technical Format: color.

If this paradigm is correct, other observations relating to this response should fall into place. Specifically, loss of macular thinning could represent hyperperfusion injury due to breach of the upper threshold of autoregulation with edema formation as has been reported by others in the eye [27, 28], and reported on MR imaging of the brain in 10–20% of women with PE [5, 6] and pathologic examination of the brain in all women dying of eclampsia [29]. One of the defining characteristics of PRES is that it can take several weeks to months for edema to resolve [4, 11]. In our Case 2 (Supplementary Data), it took 30 weeks from onset of capillary leak at 8 weeks gestation to see confluent macular thinning of both retinae at delivery, then another 15 weeks to reach baseline non-pregnant thicknesses. Macular edema persisted beyond 18 weeks postpartum in at least 2 other cases in our study. One case report in the literature also documents a lengthy recovery for edema in the eye [28]. Hypoperfusion injury is thinning in the face of a lower hemodynamic load, most commonly hypotension from peripartum hemorrhage. When this is tightly linked to the hemodynamic insult in a circulation known to autoregulate, as it was in Case 1 (Supplementary Data), there is only one explanation and that is breach of the lower threshold of effective perfusion. Finally, chronic hypertension has been shown in animal studies to shift autoregulation to the right [30, 31], and this rightward shifting would be expected to protect end-tissues from hyperperfusion injury at high systemic blood pressures (Fig. 2). Evidence for right-shifting of autoregulation in women with pre-existing hypertension is the 11 mmHg greater mean treated MAP at loss of macular thinning in those with HDP superimposed on cHTN compared to women with de novo HDP.

It must be emphasized 7 of 15 women with HDP superimposed on chronic hypertension did not develop retinal edema despite possessing some of the highest systemic blood pressures in our study. Clearly, other factors contribute to blood pressure elevations. These could include an expanded circulating blood volume; kidney disease was 3–4 times more prevalent in this cohort compared to HDP with evident capillary leak. Also, 3 of 4 women with very poorly controlled hypertension at inception might possess a right-shifted end-tissue response. These factors explain why systemic blood pressures rise in women with complex pre-existing hypertension.

Ours is the only study to prospectively follow non-hypertensive and treated hypertensive participants through a hypertensive crisis. Its focus and strengths rest on the notion that a measure of microvascular response in a relevant end-tissue such as the eye may bring greater understanding of the mechanism(s) and timing of vascular injury in severe hypertension. New findings are that vasogenic edema of the eye is more common in both PE and GHTN (74%) than previously reported [3–6]. Second, women with de novo HDP develop capillary leak at a normal systemic blood pressure. Thus, hyperperfusion/hyperfiltration is likely the initial cause of injury to the microcirculation in pregnancy. Third, the eye can be a suitable surrogate for the study of the microcirculation of brain, with limitations. Indeed, if breach of autoregulation is occurring in the eye, it could easily be occurring in the brain. We propose that subclinical edema of the brain causes pressures to rise within the cranial vault. Cerebral ischemia then leads to sympathetic activation [32], thereby driving systemic blood pressures higher and higher in order to maintain effective perfusion of that organ. The same occurs in superimposed HDP only at higher thresholds for breach of autoregulation. This supports clinical findings that hypertension in preeclampsia recurs despite effective treatment; lowering systemic blood pressure simply drives the neurogenic cascade.

There are other conditions that affect the microcirculation of the eye that can be associated with pregnancy. These include central serous chorioretinopathy, retinal occlusive disorders such as branch retinal artery and vein thrombosis and choroidal neovascularization [33]. Central serous chorioretinopathy is the most prevalent of these—seen in ~1–2% of all pregnancies, usually with severe elevations in systemic blood pressure. It is caused by choroidal ischemia leading to injury of the retinal pigment epithelium and disruption of the outer blood retinal perfusion barrier. However, all of these are easily recognized on funduscopic examination and, of course, SD-OCT.

The weaknesses of our study are the small sample sizes and the diversity of participants in both hypertensive and non-hypertensive cohorts. Issues surrounding sample size were substantially offset by our repeated measures approach; regression analyses independently confirm the findings of the stratified analyses. There were no major confounding variables. However, given the heterogeneity of our control and HDP cohorts, it is unlikely these would be reliably identified. The prospective observational cohort design is both a strength and a weakness. This is a “real life study” of high-risk hypertensive pregnancy. However, over-sampling did occur in patients with high care needs. Thus, further studies are needed to assure the validity these findings. Criticisms relating to diversity of the cohort are legitimate. However, the fact macular thinning is consistently observed in every healthy eye of every individual with or without major co-morbidities suggests the response is integral to continuing microvascular health. This is to be expected with autoregulation. There are also questions relating to the need and veracity of a non-pregnant baseline measurement. A non-pregnant baseline was needed to reveal the full extent and chronology of the macular response. However, loss of thinning, hypoperfusion injury and loss-of gain in autoregulation can all deduced by interval change on sequential images without the need for a baseline image. Finally, it might have helped to include other measures of CNS injury such as MRI of the brain to validate our findings. Given MRI has much lower spatial resolution than OCT and misses vasogenic cerebral edema in children with a “crowded cranium”, and in many established cases of preeclampsia and eclampsia [34, 35], it is unlikely this would have provided further clarity on this matter. Unfortunately, clinical decisions regarding disposition and treatment of patients with hypertensive emergency are often based on computed tomography, which has even lesser certainty.

In summary, the neurosensory retina of the eye thins at <20 weeks gestation in human pregnancy. Macular thinning is a predictable downstream effect of vasoconstriction of the microcirculation of the central retinal artery of the eye due to high flow rates and circulating blood volume in pregnancy. Thinning is lost in mid to late pregnancy in many women who go on to develop HDP. This loss of thinning can occur at a normal systemic blood pressure suggesting breach of autoregulation is due to high flow rates and hyperperfusion injury. These observations help to explain the mechanisms of blood pressure elevation in pregnancy and hypertensive emergency. A brief section on what to take away from this study appears at the end of Supplementary Data.

## Supplementary information

Final Supplemental Data - Clean
