# Supplementary material for: Sequential measurement of the neurosensory retina in hypertensive disorders of pregnancy: a model of microvascular injury in hypertensive emergency
Source: J Hum Hypertens. 2021 Oct 8;37(1):28–35. doi: 10.1038/s41371-021-00617-1 (PMC9831929; doi:10.1038/s41371-021-00617-1)
Supplement: Supplementary file 1 — Final Supplemental Data - Clean [file 41371_2021_617_MOESM1_ESM.pdf]

STRUCTURE AND HEMODYNAMICS OF THE NEUROSENSORY RETINA

Figure S1. Structure and Hemodynamics of the Neurosensory Retina

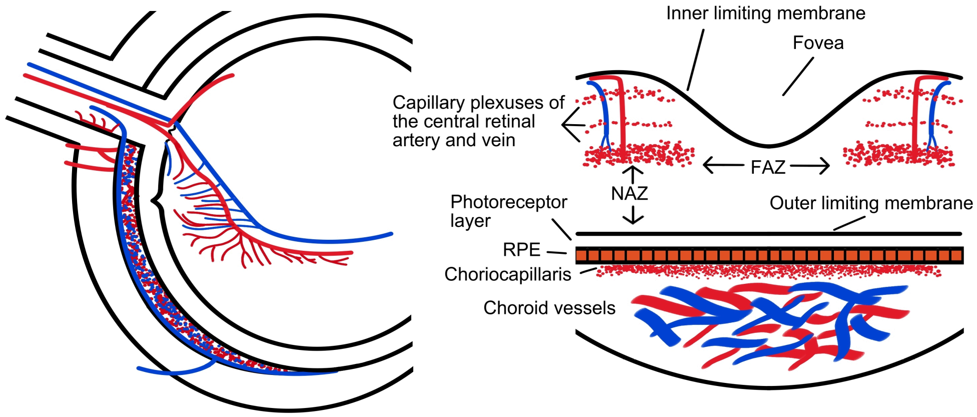

Legend: The schematic on the left is a horizontal cross-section of the right eye viewed from above showing the optic nerve and parts of the central retinal artery and vein entering through the optic nerve head, and the three major layers that comprise the eye; namely, the neurosensory retina, the choroid and the sclera. The circulation of the choroid arises from the ophthalmic vessels as small perforating branches passing directly through the sclera at the back of the eye. The fovea is represented by a small indentation in the neurosensory retina located approximately 2 disc-diameters temporal to the optic nerve head. The schematic on the right is an enlargement of the fovea with the vitreous body above and the 10 layers of the neurosensory retina from the retinal nerve fiber layer (adjacent to internal limiting membrane) to the outer photoreceptor layer. Below this are the capillaries of the choroid, the so-called the choriocapillaris, followed by the choroid vessels and outer sclera. Note that large areas of the neurosensory retina are completely avascular.

Abbreviations: FAZ – foveal avascular zone; NAZ – neurosensory avascular zone; RPE – retinal pigment epithelium

The neurosensory retina is embryologically an extension of the brain and possesses a circulation homologous in many respects to that of brain, including the presence of blood-retinal barriers and the ability to autoregulate[1]. Water, oxygen and other small molecules enter the retina via paracellular pathways that surround endothelial cells and their supporting glycocalyx. Access is flow dependent and unconstrained, and once inside these move freely throughout the retina according to the principles of simple diffusion[2]. Water, solute, nutrient, albumin and other larger molecules enter and leave the neurosensory retina via transcytosis by neuroglia, principally the retinal Müller glia, whose size bridges the entire axial dimension of the tissue. Both the paracellular and transcellular pathways are highly integrated and regulated. However, substances moving through glia often possess significant osmotic effects and so their movements must be constrained, as their presence within the interstitium may alter overall movement of water and oxygen.

Each eye has 2 separate circulations, yet large areas of the retina are completely avascular. The neurosensory retina interrogated by Spectral Domain-Optical Coherence Tomography (SD-OCT) effectively resides between 2-permeability barriers[2]. Water enters the eye by crossing the inner blood-retinal barrier at 3 capillary plexuses, and together with contributions from the glia and other sources forms the Inward Paracellular Current. The rate and magnitude of this is largely controlled by autoregulation of the central retinal artery. Larger molecules 'ride' this

current and thereby support the metabolic and signaling needs of attendant tissues. Water and oxygen also enter from the choroid circulation at the back of the eye to sustain the retinal pigment epithelium (RPE) and photoreceptors layers. Movement of water out of the neurosensory retina is controlled by a high osmotic gradient for both chloride ion and lactate generated by the photoreceptor layers and pumped by transepithelial transporters on the cells of the RPE to its outward-facing side. This drives the Outward Paracellular Current. The tight junctions of the RPE and outer limiting membrane (collectively known as the outer blood-retinal barrier) maintain these gradients and thereby the directional movement of water towards the back of the eye. Fenestrated capillary walls and high onchotic pressures for albumin within the choriocapillaris and choroid vessels facilitate recovery of interstitial fluid into the systemic circulation.

Given the above, certain generalizations pertain. First, the convective movement of water from feeder capillaries of the central retinal artery is a major determinant of the Inward Paracellular Current. Since these tissues reside within the fibrous capsule of the eye, pressure within the interstitial fluid space, together with other water bodies in the eye are a second determinant of paracellular flow. If intraocular pressure is allowed to rise, capillary perfusion pressure will eventually become insufficient to sustain effective blood flow to the retina. Third, breach of either of the 2 blood-retinal barriers represents a major insult to the retina. It affords sudden, and uncontrolled entry of both solute and protein, which then secondarily alters water movement and perfusion dynamics within the neurosensory retina. Also, proteins are injurious to the photoreceptor layers. Protein is removed from the neurosensory retina by transcytosis via retinal Müller glia, a process that can take months to restore. Over this time frame, poor flow, ischemia and the presence of these proteins initiate an inflammatory response and tissue repair potentially further damaging permeability barriers[2]. Finally, the outer blood retinal barrier at the RPE has greater permeability than the inner blood retinal barrier[3]. A more permeable barrier does not imply greater propensity to breach. Rather, this facilitates perfusion of the outer retina during periods of relative hypoperfusion by the inner retinal circulation.

The Starling equation that defines net paracellular transudation predicts that increases in blood flow and circulating blood volume that peak in mid to late pregnancy, if unopposed, could lead to over-filtration and flooding of tissues, including the brain and retinae[4]. The fact the retina thins in pregnancy and is most thinned at the time of maximal hemodynamic load on the maternal circulation signals the presence of a mitigating response. No one has directly measured interstitial fluid pressures within the neurosensory retina. However, it is posited these pressures are very low, if not negative, due to the putative strength of the Outward Paracellular Current needed to draw fluid across large, avascular areas within this tissue compartment[2]. If true, gain-of-function of autoregulation of the central retinal artery thereby limiting transudation of water across the inner blood-retinal barrier could cause the neurosensory retina to thin. Thus, measurement of this unique tissue in pregnancy may reflect the action of the autoregulatory reflex on the microcirculation of the eye.

References for Structure and Hemodynamics of The Neurosensory Retina

1. Kur J, Newman EA, Chan-Ling T. Cellular and physiological mechanisms underlying blood flow regulation in the retina and choroid in health and disease. Prog Retin Eye Res. 2012; 31:377-406.
2. Daruich A, Matet A, Moulin A, Kowalczuk L, Nicolas M, Sellam A, et al. Mechanisms of macular edema: beyond the surface. Prog Retin Eye Res. 2018; 63:20-68. doi:10.1016/j.preteyeres.2017.10.006.
3. Peng S, Rao VS, Adelman RA, Rizzolo LJ. Claudin-19 and the barrier properties of the human retinal pigment epithelium. Invest Ophthalmol Vis Sci. 2011; 52:1392-1403.
4. Kimelberg HK. Water homeostasis in the brain: basic concepts. Neuroscience. 2004; 129:851-860.

SD-OCT FINDINGS OF HYPOPERFUSION INJURY IN HYPERTENSIVE DISORDERS OF PREGNANCY

Case 1 had a twin pregnancy and developed the HELLP syndrome at 29 weeks gestation. Her macular thickness profile on the Early Treatment of Diabetic Retinopathy Study[1] (ETDRS) grid and mean arterial pressure (MAP) at sequential clinical encounters are shown in the upper panel of Figure S2. At 15 weeks gestation, both maculae show the thinning response characteristic of healthy eyes in early pregnancy. At 29 weeks gestation, partial effacement of that thinning is seen on the cumulative change profile to the left of MAP. This is broadly confluent and widespread when viewed on the interval change profile to the right of MAP. At 34 weeks gestation, her pregnancy is further complicated by a small placental abruption (estimated blood loss 500cc) with fetal distress and the baby is delivered by emergency cesarean section. Post-operative blood pressures fall from 140/90 mmHg at triage the day before to 113/70 mmHg

Figure S2. Macular Thickness Change and MAP in 2 Women with HDP and Hypoperfusion Injury

Gestation	sf	is	in	ii	it	os	on	oi	ot	sf	is	it	ii	in	os	ot	oi	on	MAP	sf	is	in	ii	it	os	on	oi	ot	sf	is	it	ii	in	os	ot	oi	on
15 wks	-6	-10	-7	-6	-7	-8	-5	-1	-6	-5	-7	-6	-4	-5	-5	-5	-1	-3	81	-6	-10	-7	-6	-7	-8	-5	-1	-6	-5	-7	-6	-4	-5	-5	-5	-1	-3
29 wks	-1	-4	-2	0	-2	-4	-1	2	-3	-2	-5	-4	0	1	-5	-4	0	0	82	5	6	5	6	5	4	4	3	3	3	2	2	4	6	0	1	1	3
Del 34 wks	-10	-12	-9	-7	-11	-10	-7	-5	-8	-10	-10	-11	-6	-9	-10	-9	-4	-4	84	-9	-8	-7	-7	-9	-6	-6	-7	-5	-8	-5	-7	-6	-10	-5	-5	-4	-4
3 wks	-5	-5	-2	-2	-4	-6	-2	0	-4	-6	-6	-6	-2	-3	-8	-7	-2	-1	101	5	7	7	5	7	4	5	5	4	4	4	5	4	6	2	2	2	3
23 wks	0	0	0	0	0	0	0	0	0	0	0	0	0	0	0	0	0	0	81	5	5	2	2	4	6	2	0	4	6	6	6	2	3	8	7	2	1

Gestation	sf	is	in	ii	it	os	on	oi	ot	sf	is	it	ii	in	os	ot	oi	on	MAP	sf	is	in	ii	it	os	on	oi	ot	sf	is	it	ii	in	os	ot	oi	on
10 wks	1	3	5	3	1	2	5	3	3	0	3	2	0	-2	3	1	3	4	122	1	3	5	3	1	2	5	3	3	0	3	2	0	-2	3	1	3	4
14 wks	1	3	3	1	0	1	3	5	1	1	4	3	2	0	4	4	6	6	106	0	0	-2	-2	-1	-1	-2	2	-2	1	1	1	2	2	1	3	3	2
19 wks	1	3	2	3	0	2	4	7	1	0	3	2	0	-1	-1	0	2	0	97	0	0	-1	2	0	1	1	2	0	-1	-1	-1	-2	-1	-5	-4	-4	-6
25wks	-3	-2	-2	-2	-1	0	1	3	1	-4	-2	-3	-5	-4	-2	-2	0	-3	93	-4	-5	-4	-5	-1	-2	-3	-4	0	-4	-5	-5	-5	-3	-1	-2	-2	-3
33 wks	-1	0	1	2	1	2	0	1	1	-4	-2	-3	-3	-4	0	0	2	2	98	2	2	3	4	2	2	-1	-2	0	0	0	0	2	0	2	2	2	5
35 wks	-1	-1	1	0	-1	0	3	5	1	-2	-2	-2	-4	-4	0	2	2	0	102	0	-1	0	-2	-2	-2	3	4	0	2	0	1	-1	0	0	2	0	-2
37 wks	0	0	0	-1	-1	1	2	1	1	-4	-2	-4	-5	-5	-2	-3	-1	-1	108	1	1	-1	-1	0	1	-1	-4	0	-2	0	-2	-1	-1	-2	-5	-3	-1
Del 38 wks	-5	-5	-7	-5	-5	-2	-4	0	-3	-6	-4	-5	-7	-8	-4	-3	0	-4	115	-5	-5	-7	-4	-4	-3	-6	-1	-4	-2	-2	-1	-2	-3	-2	0	1	-3
6 d	-3	-1	-2	0	1	0	0	1	1	-3	-2	-2	-4	-4	-2	-1	-1	-1	103	2	4	5	5	6	2	4	1	4	3	2	3	3	4	2	2	-1	3
5 wks	-4	-2	-2	-2	-3	-2	-2	-1	-3	-7	-4	-3	-4	-5	-3	-7	-4	-1	99	-1	-1	0	-2	-4	-2	-2	-2	-4	-4	-2	-1	0	-1	-1	-6	-3	0
9 wks	-1	-1	-3	-2	0	-2	-4	-2	-2	0	-1	-1	-2	-2	-4	-3	-3	-3	112	3	1	-1	0	3	0	-2	-1	1	7	3	2	2	3	-1	4	1	-2
15 wks	-2	-2	-2	-2	-2	-1	-3	-2	-1	-1	-3	-3	-4	-3	-5	-4	-4	-6	119	-1	-1	1	0	-2	1	1	0	1	-1	-2	-2	-2	-1	-1	-1	-1	-3
61 wks	0	0	0	0	0	0	0	0	0	0	0	0	0	0	0	0	0	0	99	2	2	2	2	2	1	3	2	1	1	3	3	4	3	5	4	4	6

Legend: The upper panel of this Figure represents macular thickness change and MAP over the course of pregnancy for Case 1. Corresponding data for Case 2 appears below Case 1. Gestational age at image acquisition appears along the left margin of the Figure. MAP and its values at each gestational encounter appear in the bright yellow band running down the center of the Figure. ETDRS profiles to the left of MAP represent cumulative change in macular thickness at the time of measurement. ETDRS profiles to the right of MAP represent interval change (i.e., compared to thicknesses at the gestational encounter immediately

preceding it). Lower case lettering across the top of each participant's series of measurements identify the 9 segments of the ETDRS grid for the right eye in pale green and for the left eye in pale cyan blue. Pink signifies the subfoveal segment of each eye. Numbers populating the table indicate change in macular thickness for that segment at each gestational encounter. Zeros across the last postpartum row in each participant's cumulative profile indicate this to be the referent non-pregnant baseline. Pale green, pale cyan blue and pink colorations with black numbering indicate no significant difference from the referent measurement using the clinical decision support tool. Deep cyan blue with white numbering represents statistically significant macular thinning. Grey coloration with black numbering represents all other segments in that series (both eyes) possessing the same sign ('–' for thinning). Orange and pale orange coloration with white/black numbering are analogous designations for significant loss of macular thinning. Note, change in macular response (from thinning to loss of thinning, or vice versa) is best visualized on the interval change profiles, whereas the overall dimension of the tissue (thinned or near baseline thickness) is best appreciated in the cumulative change profile. Two additional color schema are used to designate other behaviors of the retina; this is only shown in the interval change profiles. When macular thinning occurs in the context of a lower systemic blood pressure, particularly if associated with postpartum hemorrhage, we consider this to be definitive evidence of hypoperfusion injury to that eye. Macular thinning with blood pressure lowering following a hypertensive event is equally concerning as this may represent iatrogenic hypoperfusion. Coloration in both of these instances is changed from deep cyan and grey to royal blue and wine. Also, transient loss of thinning at lower systemic blood pressures while pregnant (coloration changed to chocolate brown and tan) has several potential explanations, both normal and abnormal. For further discussion on this matter see 'What to Take Away from this Study' at the end of Supplemental Data.

Abbreviations: MAP – mean arterial pressure in mmHg; HDP- hypertensive disorder(s) of pregnancy; Gestation – gestational age; sf – subfoveal segment; is – inner circle superior segment; in – inner circle nasal segment; ii – inner circle inferior segment; it – inner circle temporal segment; os – outer circle superior segment; on – outer circle nasal segment; oi – outer circle inferior segment; ot – outer circle temporal segment; wks – weeks gestation or weeks postpartum; Del – delivery; d – days post delivery.

(MAP 84 mmHg). This *delta* MAP is only -17 mmHg, yet retinae now show new findings of extensive, confluent thinning. By definition, this is hypoperfusion injury (i.e., macular thinning at lower hemodynamic load associated with a peripartum hemorrhage). Note also, absolute MAP is slightly higher than 82 mmHg measured 5 weeks earlier, and 81 mmHg measured 19 weeks earlier at 15 weeks gestation. The explanation for hypoperfusion at such modest changes in absolute and *delta* MAP relates to the antecedent finding of loss of thinning. If loss of thinning is due to retinal edema to the extent that it causes intraocular pressures to rise, this alters the relationship between perfusing pressure and intraocular pressure such that previously tolerated systemic blood pressures may be insufficient to adequately perfuse the organ.

Case 2 presented to her family physician at 8 weeks gestation with severe, untreated hypertension and a blood pressure of 240/120 mmHg. She was reported to be asymptomatic, but did endorse influenza-like symptoms the week before. She was hospitalized and stabilized on treatment with methyldopa and labetalol with blood pressures falling to 156/105 mmHg (MAP 122 mmHg) by the time of enrolment. Her ETDRS profile and MAP are shown in the lower panel of Figure S2. Note, her cumulative change profile from 10 weeks through 19 weeks gestation shows patchy thickening of both maculae. This is vasogenic edema of the eye from Posterior Reversible Encephalopathy Syndrome (PRES), as reported by others[2,3]. Note, MAP at 14 weeks gestation is 106 mmHg and with this 16-mmHg reduction in systemic blood pressure there appears be slight loss of thinning of her left macula. This is not statistically significant at this juncture. There are a couple of explanations, including loss-of-gain of autoregulation as the central retinal artery dilates in order to maintain effective perfusion of her edematous retina. Since systemic blood pressures were still high, the dosage of her anti-hypertensive medication was increased and at each of the 2 subsequent encounters blood pressures fall to even lower levels. Now her maculae are thinning while blood pressure is declining, meaning this is hypoperfusion injury and the preceding change at 14 weeks was truly early loss-of-gain. Blood pressures subsequently recover with both retinae exhibiting modest thinning (a normal response) by delivery at 38 weeks gestation. However, postpartum MAP drops again below 105 mmHg, and the above sequence is repeated with loss-of-gain progressing to hypoperfusion injury at 5 weeks postpartum. This observation is particularly important because hypoperfusion is iatrogenic due to overly aggressive lowering of the systemic blood pressure. Moreover, it happened twice at the same systemic blood pressure.

Due to the presence of pre-existing PRES, Case 2's profile could only be reliably interpreted following collection of the final postpartum image.

References For SD-OCT Findings Of Hypoperfusion Injury In HDP

1. Huang J, Liu X, Wu Z, Xiao H, Dustin L, Sadda S. Macular thickness measurements in normal eyes with time domain and fourier domain optical coherence tomography. Retina. 2009; 29:980-987.
2. Neudorfer M, Spierer O, Goder M, Newman H, Barak S, Barak A, et al. The prevalence of retinal and optical coherence tomography findings in preeclamptic women. Retina. 2014; 34:1376-1383.
3. Theodossiadis PG, Kollia AK, Gogas AK, Panagiotidis D, Moschos M, Theodossiadis GP. Retinal disorders in preeclampsia studied with optical coherence tomography. Am J Ophthalmol. 2002; 133:707-709.

ABSOLUTE MACULAR THICKNESSES AND REGRESSION ANALYSES

Table S1 and Table S2 are analogous to Tables 2 and 3 in the main manuscript, excepting that they are populated by measures of absolute macular thickness and their respective contrasts. These data are provided for completeness.

Table S1. Absolute Macular Thickness (µm) at Baseline and <20wks Gestation

Type of Pregnancy	Baseline Mean (95%CI)	<20wks Gestation Mean (95%CI)	*P*–Value vs Baseline
**HDP (n=27)**	295.4 (290.7, 300.2)	291.2 (286.5, 296.0)*	<0.001
**Controls (n=11)**	295.5 (288.0, 302.9)	291.6 (284.2, 299.1)	<0.001

Legend: * *P*=0.370 compared to controls

Abbreviations: µm – micrometers; wks – weeks; CI – confidence interval; vs – versus; *P*-value – probability threshold to indicate statistical significance; HDP – hypertensive disorder(s) of pregnancy

Table S2. Absolute Macular Thickness (µm) from <20wks to Delivery in Controls and HDP

Type of Pregnancy	At Baseline Mean (95%CI)	At <20wks Mean (95%CI)	*P*–Value vs Baseline	At 20-40wks* Mean (95%CI)	At Delivery Mean (95%CI)	*P*–Value vs <20wks
**De novo HDP (n=12)**	297.9 (290.5, 305.4)	292.8 (285.3, 300.2)	<0.001	294.5 (287.1, 302.0)	294.1 (286.6, 301.6)	0.001
**HDP/cHTN w/o LOT (n=7)**	295.5 (289.6, 301.4)	291.9 (286.0, 297.9)	<0.001	291.7 (285.8, 297.7)	291.1 (285.1, 297.0)	0.001
**HDP/cHTN with LOT (n=8)**	291.6 (282.0, 301.1)	288.1 (278.6, 297.7)	<0.001	288.8 (279.2, 298.3)	290.3 (280.8, 299.9)	<0.001
**Controls (n=11)**	295.5 (288.0, 303.0)	291.8 (284.3, 299.2)	<0.001	292.2 (284.8, 299.7)	290.7 (283.2, 298.2)	<0.001

Legend: * 20-40 weeks means from 20 weeks gestation up to, but not including delivery, as delivery was expedited in all but 1 woman with HDP

Abbreviations: µm – micrometers; wks – weeks gestation; HDP – hypertensive disorder(s) of pregnancy; *P*-value – probability threshold to indicate statistical significance; vs – versus; CI – confidence interval; cHTN – chronic hypertension; HDP/cHTN – HDP superimposed on cHTN; w/o – without; LOT – loss of thinning

Note, findings in total macular thickness are essentially the same as those provided in Tables 2 and 3 in the main manuscript for change in macular thickness between the various cohorts.

Regression Analyses

Table S3 is a regression analysis of the category of HDP (i.e., non-hypertensive controls, de novo HDP and HDP superimposed on chronic hypertension with and without loss of thinning) and gestational age (baseline, <20 weeks gestation, from 20 weeks up to, but not including delivery and at delivery) against baseline macular thickness in non-hypertensive controls.

Table S3. Regression of HDP Category and Gestational Age Against Macular Thickness

Group	Estimated Value (95%CI)	*P*-value
Baseline Thickness in Non-hypertensive Controls **(Intercept)**	295.5 (288.1, 302.8)	-
Baseline Thickness in De novo HDP	297.9 (290.9, 305.0)	0.636
Baseline Thickness in Superimposed HDP without loss of thinning	295.5 (286.3, 304.7)	0.996
Baseline Thickness in Superimposed HDP with loss of thinning	291.6 (282.9, 300.2)	0.506
Thickness at Gestational Age <20wks in Controls	291.7 (284.4, 299.1)	0.000
Thickness at Gestational Age 20wks to Delivery in Controls	292.3 (285.0, 299.7)	0.000
Thickness at Delivery in Controls	290.7 (283.3, 298.0)	0.000
Thickness at Gestational Age <20wks in de novo HDP	292.8 (285.8, 299.8)	0.002
Thickness between 20wks and Delivery in de novo HDP	294.5 (287.5, 301.5)	0.496
Thickness at Delivery in de novo HDP	294.1 (287.0, 301.1)	0.040
Thickness at <20wks in Superimposed HDP without loss of thinning	291.9 (282.7, 301.1)	0.711
Thickness from 20wks - Delivery in Superimposed HDP without loss of thinning	291.7 (282.5, 300.9)	0.181
Thickness at Delivery in Superimposed HDP without loss of thinning	291.1 (281.9, 300.3)	0.430
Thickness at <20wks in Superimposed HDP with loss of thinning	288.3 (279.6, 296.9)	0.372
Thickness from 20wks to Delivery in Superimposed HDP with loss of thinning	288.8 (280.2, 297.4)	0.422
Thickness at Delivery in Superimposed HDP with loss of thinning	290.0 (281.4, 298.7)	0.000

Abbreviations: CI – Confidence Interval; HDP – Hypertensive Disorders of Pregnancy.

Baseline macular thicknesses in HDP cohorts were not significantly different from baseline thickness in non-hypertensive controls. Modest, but significant macular thinning was observed at all gestational intervals in non-hypertensive controls. The same occurred at <20 weeks gestation in de novo HDP (P=0.002), but transitioned to loss of thinning by delivery (P=0.040). Early pregnancy thinning was present, but muted and not significant at <20 weeks gestation in HDP superimposed on chronic hypertension (also seen in Table S5 on analyses of the ganglion cell and inner plexiform layers in Supplemental Data: Fractional Thinning). Late pregnancy loss of thinning was significant at delivery (P<0.001) in HDP superimposed on chronic hypertension in those where loss of thinning was observed. Thus, gestational age and HDP subtype were the main determinants of change in macular thickness in our study.

Race[1,2], aging, sex, and axial length of the eye (not measured in our study)[3-5] are known to be associated with differences in macular thickness. Since the effects of these variables are long-term and not reversible, it is unlikely they would be responsible for macular thinning or loss of

thinning in pregnancy. Eight participants in our study were of non-Caucasian descent, of which 2 were controls, 3 went on to develop de novo HDP and 3 had HDP superimposed on chronic hypertension. Baseline macular thickness in these 9 women was on average 9 µm thinner (mean thickness 288 µm; 95%CI 281, 295) than Caucasian women. However, the extent of macular thinning in pregnancy was the same in non-Caucasian and Caucasian women (4 µm; 95%CI 6, 2 versus 5 µm; 95%CI 6, 4; $P>0.05$). There were too few non-Caucasians to look at the effects of group and gestational age on this variable.

Maternal age was examined, but again study participants were young and the period of observation too short to have any meaningful role on macular response ($P=0.365$). Interestingly, participants with chronic kidney disease (n=6) had on average 20 µm thicker maculae ($P=0.011$) than other pregnant women in our study. This observation finds support from a longitudinal study of the effects of proteinuria and moderately reduced glomerular filtration rate on macular thickness in 7709 Japanese patients with diabetes mellitus followed over 5.6 years[6]. Women with chronic inflammatory conditions, including rheumatoid arthritis, lupus and crohn's disease (n=5) had average 20 µm thinner maculae ($P=0.029$). At least 1 of these participants was on hydroxychloroquine during her incident pregnancy, or had received this medication previously. This could have contributed to the finding.

No other confounding variables were found to impact the main findings. This independent analysis of the entire data set mirrors and supports the findings of the stratified analyses in Tables 3 and S2.

References For Absolute Macular Thickness and Regression

1. Kelty PJ, Payne JF, Trivedi RH, Kelty J, Bowie EM, Burger BM. Macular thickness assessment in healthy eyes based on ethnicity using Stratus OCT optical coherence tomography. Invest Ophthalmol Vis Sci. 2008; 49:2668-2672.
2. Duan XR, Liang YB, Friedman DS, Sun LP, Wong TY, Tao Qs, et al. Normal macular thickness measurements using optical coherence tomography in healthy eyes of adult Chinese persons: the Handan Eye Study. Ophthalmology. 2010; 117:1585-1594.
3. Myers CE, Klein BEK, Meuer SM, Swift MK, Chandler CS, Huang Y, et al. Retinal thickness measured by spectral-domain optical coherence tomography in eyes without retinal abnormalities: the Beaver Dam Eye Study. Am J Ophthalmol. 2015; 159:445-456.
4. Subhi Y, Forshaw T, Sorensen TL. Macular thickness and volume in the elderly: a systematic review. Aging Res Rev. 2016; 29:42-49.
5. Yamamoto M, Fujihara K, Ishizawa M, Osawa T, Kaneko M, Ishiguro H, et al. Overt proteinuria, moderately reduced eGFR and their combination are predictive of severe diabetes retinopathy or diabetic macular edema in diabetes. Invest Ophthalmol Vis Sci. 2019; 60:2685-2689.

FRACTIONAL THINNING OF VASCULARIZED AND NON VASCULARIZED REGIONS, INCLUDING THE INTERNAL AND EXTERNAL RETINA AND CHOROID

Table S4 profiles absolute and fractional macular thinning in pregnancy in the subfoveal field, and at the inner 1-3 mm and outer 3-6 mm circles of the ETDRS grid from baseline to delivery in non-hypertensive controls and superimposed HDP where thinning was maintained up to and including delivery. We surmised that since thinning is a regulated response, it should be consistent across the entire macula in these individuals. Absolute thickness change appearing in the 3 columns to the left of the Table is larger and easier to identify, but conceal the fact that proportional change is highly uniform across the entire macula. Note also, thinning in avascular regions like the fovea is equivalent to thinning in more vascularized tissues like the inner and outer circles. This suggests the presence blood within vessels in these regions has little to do with the macular thinning response. Fractional thinning at the periphery of the ETDRS grid is slightly less ($P<0.05$) and likely due to weakened draw from the outward paracellular current at locations more distant from the center of the macula.

Table S4. Absolute Versus Relative Change in Macular Thickness in Non-hypertensive Pregnant Controls and Women with HDP

**Controls**	**Absolute Thickness Change (µm)**			**Fractional Change (% Baseline Thickness)**		
	**Subfoveal**	**Inner Circle**	**Outer Circle**	**Subfoveal**	**Inner Circle**	**Outer Circle**
**Mean**	**-4.8**	**-6.7**	**-3.6**	**-2.0**	**-2.1**	**-1.3**
**95%CI**	**(-5.4,-4.2)**	**(-7.0,-6.3)**	**(-4.0,-3.3)**	**(-2.2,-1.7)**	**(-2.2,-2.0)**	**(-1.4,-1.2)**
**HDP**	**Absolute Thickness Change (µm)**			**Fractional Change (% Baseline Thickness)**		
	**Subfoveal**	**Inner Circle**	**Outer Circle**	**Subfoveal**	**Inner Circle**	**Outer Circle**
**Mean**	**-4.5**	**-5.8**	**-2.9**	**-1.7**	**-1.8**	**-1.0**
**95%CI**	**(-5.2,-3.7)**	**(-6.1,-5.5)**	**(-3.2,-2.6)**	**(-2.0,-1.4)**	**(-1.9,-1.7)**	**(-1.1,-0.9)**

Abbreviations: HDP – hypertensive disorders of pregnancy; CI – Confidence Interval

The Internal and External Retina

Blood within the central retinal artery and vein supply the superficial capillary plexus, which resides within the ganglion cell layer (GCL) and inner plexiform layer (IPL) of the internal retina. The deep capillary plexus extends to the inner nuclear layer. There are no capillaries below the inner nuclear layer; the deeper layers of the neurosensory retina are completely avascular.

Together, the GCL and IPL comprise an ellipsoid, ‘donut’-shaped structure fully within the macula with its minor 4 mm axis oriented in the vertical plane and its major axis, almost 5 mm in diameter, in the horizontal plane centered at the fovea. Table S5 shows the extent of pregnancy-induced thinning at the GCL/IPL and in the residual macular segments extending from the inner nuclear layer to the retinal pigment epithelium. Again, we focused in non-hypertensive controls and HDP superimposed on chronic hypertension where thinning was maintained up to and including delivery as this is more likely to be consistent within tissues. However, we also examined thinning in women with de novo HDP and HDP superimposed on chronic hypertension, but only at gestational intervals that preceded loss of thinning. The purpose of this was to see whether we might localize an earlier event that preceded loss of thinning, and if so, whether this localized to the inner or outer retina.

Table S5. Mean Fractional Change (%) in Baseline Thickness During Thinning in the GCL/IPL and Residual Layers in Non-hypertensive Pregnancy and Women with HDP

Type of Pregnancy	GCL/IPL Mean (95%CI)	Residual Segments Mean (95%CI)	P–Value vs Region
**Controls (n=11)**	-2.4 (-2.9, -1.8)	-1.9 (-2.5, -1.4)	0.04
**HU (n=7)**	-2.3 (-3.0, -1.6)	-1.9 (-2.7, -1.2)	0.20
**dnHDP (n=12)**	-2.8 (-3.1, -2.4)	-1.4 (-1.7, -1.0)*	<0.001
**sH (n=8)**	-2.2 (-2.7, -1.8)	-1.4 (-1.8, -0.9)*	<0.001

Legend: * P=0.005 compared to controls

Abbreviations: GCL – ganglion cell layer; IPL – inner plexiform layer; HDP – hypertensive disorder(s) of pregnancy; CI – confidence interval; *P*-value – probability threshold to indicate statistical significance; vs – versus; Controls – non-hypertensive women attending a high-risk pregnancy clinic; HU – HDP superimposed on chronic hypertension where macular thinning in maintained up to and including delivery; dnHDP – de novo hypertension in pregnancy; sH – superimposed HDP where loss of macular thinning occurs before or at the time of delivery.

Note, fractional thinning is the same in the GCL/IPL and the residual macular layers of the macula in non-pregnant controls and women with HDP where thinning is maintained till the time of delivery. However, in women who go on to develop loss of macular thinning in pregnancy, fractional thinning in the residual macula (i.e., external retina) is proportionally half that of the GCL/IPL.

We then looked to identify those participants with potentially limited thinning, specifically residual macular thinning less than the lower threshold of the 95% confidence interval in their respective cohort. There were 10 of these in the entire cohort, including 2 non-hypertensive pregnant controls, 3 participants with HDP superimposed on chronic hypertension and 5 participants with de novo HDP. Fractional thinning in the GCL/IPL layers in these individuals was significantly greater (-3.3; 95%CI -3.6,-2.9) than any of the cohorts in Table S5. However, 2 of the 3 with HDP superimposed on chronic hypertension had reduced thinning in both the GCL/IPL and residual macular segments. These are very limited data and do not support any conclusion. We provide these details simply to be complete and to assist those who may wish to investigate this matter further.

Together, these observations support the notion that the neurosensory retina thins broadly and confluently throughout its dimension during pregnancy and this is due to widespread constriction of the central retinal artery, akin to an autoregulatory response. The outer limiting membrane is the only diffusion barrier within the neurosensory retina[1], and is not a high restriction barrier like the blood retinal barriers described above. Its purpose is simply to limit the passage of larger molecules like proteins from the internal retina to the more sensitive outer photoreceptor layers. Therefore, if the overall hydration status of the neurosensory retina changes, the effects of this appear to be uniform across the entire tissue.

The Choroid

Several groups have focused on the choroid circulation in normal pregnancy and preeclampsia. A recent systematic review and meta-analysis of 14 studies comprising 1,227 participants[2] reported that the choroid thickens in normal pregnancy compared to non-pregnant

controls and the choroid in preeclampsia was thinner compared to non-pregnant controls in 3 studies and thicker in 1 study. This resulted to no net difference in the overall analysis. All studies included in these analyses were cross-sectional studies, except for one study comparing first and third trimester choroid images in 27 normal pregnant participants with a single image in 25 age-matched controls[3]. They report the choroid is thicker at 6-8 weeks gestation than at 32-37 weeks gestation. Both mean thickness measurements in normal pregnancy were greater than the mean thickness of non-pregnant controls.

In our study, choroid thickness was estimated in both eyes from a standard 512 x 128 scanning format centered on the fovea. Measurements were obtained using an electronic caliper within the programming of the instrument as the distance between the outer boundary of the retinal pigment epithelium and the inner surface of the sclera. Since multiple paired images were obtained in our study, we were able to verify the position of the sclera in all but 1 individual.

Assuming a measurement error of ±20 μm and counting only those eyes with a non-pregnant baseline measurement, thickening was seen in early pregnancy in 12 of 21 eyes in 11 non-hypertensive controls, 6 of 14 eyes in 7 participants with HDP superimposed on chronic hypertension where macular thinning was maintained throughout pregnancy, and 8 of 20 eyes in 11 women with de novo HDP. Twenty-four eyes were thinned at the first gestational measurement (15 weeks), meaning thickening was missed due to the later gestational age at enrollment into our study, or that an early pregnancy choroid response may include thinning as well as thickening. Five eyes fell within the attributed ±20 μm measurement error. More importantly, the choroid progressively thinned in every eye as delivery approached to achieve a less thickened dimension, or more commonly, an overtly thinned choroid thickness compared to baseline measurements. Further, the extent of these thickening and thinning fluctuations of the choroid appear to be larger than previously appreciated (Table S6). Since the 2 eyes behaved similarly (paired T-test = 0.31-0.75, correlation = 0.33-0.56 in the 3 cohorts), only measurements taken from the left eye are reported.

Table S6. Mean Change in Choroid Thickness in a Single Eye in HDP and Non-hypertensive Controls

	Extent of Early Pregnancy Thickening		Extent of Late Pregnancy Thinning	
	Sample Size (No. of Eyes)	Mean (95%CI) μm	Sample Size (No. of Eyes)	Mean (95%CI) μm
Controls	(n=7)	65.9 (19.5, 112.2)	(n=11)	70.3 (38.2, 102.3)
HU	(n=3)	52.3 (18.6, 86.0)	(n=7)	83.0 (46.4, 119.6)
dnHDP	(n=4)	35.0 (11.3, 58.7)	(n=6)	54.2 (39.0, 69.3)

Abbreviations: HDP – hypertensive disorders of pregnancy; CI – Confidence Interval; No – number; HU – HDP superimposed on chronic hypertension where macular thinning continued through delivery; dnHDP – de novo HDP

Our findings concur with those of Dadaci et al[3], excepting the magnitude of these changes are 3-5 times greater likely due to selection (i.e., selection of the greatest and smallest dimensions of this tissue and selection of only those profiling this response). It remains uncertain whether loss of macular thinning in de novo HDP is reflected in choroid thickness or not, as we

also observed a number of large spontaneous fluctuations in choroidal thickness in the late stages of pregnancy, both in participants with and without loss of macular thinning (see Figure S3 for examples of this). We believe that the choroid circulation does not autoregulate and these data support that (i.e., in some participants the retina thinned as the choroid thickened and the choroid thinned as the retina thickened). Furthermore, autoregulation would likely interfere with the choroid's ability to be an effective absorption surface for the outward paracellular current, particularly in pregnancy with its inherently high-flow state.

Figure S3. Sequential Choroid Thickness Measurements where the Pattern Supports or Refutes a Concordant Relationship to Changes Occurring in the Macular

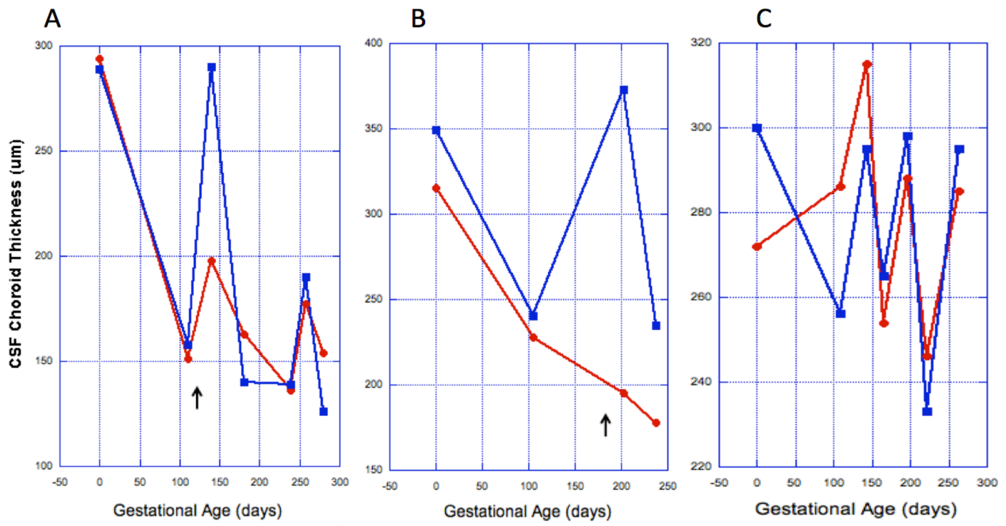

Legend: Note, the first point in these series is the baseline; the second point is a <20wk measurement. The last point is at delivery. An arrow indicates the timing of loss of macular thinning in 2 participants. Participant A had de novo gestational hypertension where loss of macular thinning was accompanied by concordant loss of thinning of the choroid in both eyes. A second spike of loss of choroid thickness appears later in the profile, but macular thickness was actually starting to thin in both eyes at that juncture. Thus, the second spike is non-concordant with macular response at that time. Participant B had early pregnancy de novo preeclampsia and HELLP Syndrome with loss of thinning of the choroid concordant with loss of thinning in the macula. However, this only occurred in the left eye, where the changes in the macula where bilateral and more severe on the right. Participant C had gestational hypertension superimposed on longstanding well-controlled hypertension on drug treatment. Her macula maintained stable thinning throughout pregnancy, yet her choroid revealed multiple spikes of thinning and thickening. At delivery, she suffered severe postpartum hemorrhage and her MAP dropped 68 mmHg from 107 to 39 mmHg. Both maculae thinned revealing hypoperfusion injury, but her choroid thickened. These findings are non-concordant and are likely related to hypoxia and the injury response.

Abbreviations: CSF – central subfoveal field; MAP – mean arterial pressure

The choroid sub-serves at least 3 important physiologic functions in the orbit. It supplies oxygen to the external retina, which is easily accomplished as it one the most vascularized tissues in the body. Secondly, the choroid is responsible for the absorption of interstitial fluid from large avascular zones within the retina and removes this water from the eye. Again, high vessel density and most likely coordinated phasic contractions/relaxations of small subfields of its vascular territory controlled by local sympathetic activity facilitate this action. Finally, the choroid may function to balance offsetting changes in other regions of the orbit[4]. We speculate that as the central retinal artery constricts in human pregnancy causing the macula to thin, interstitial volume and pressure are lowered presenting less optimal conditions for the recovery of water. These vessels are also filled with blood due to the more luxuriant flow blood and volume in pregnancy. This might explain choroid behavior in pregnancy. Dadaci attributed choroid thinning in the third trimester to increases in estrogen and progesterone and circulating concentrations of catecholamines[3]. Systemic sympathetic activation in late pregnancy is certainly

a potent stimulus for constriction of choroidal vessels[5].

References For Fractional Thinning

1. Daruich A, Matet A, Moulin A, Kowalczuk L, Nicolas M, Sellam A, etal. Mechanisms of macular edema: beyond the surface. Prog Retin Eye Res. 2018; 63:20-68. doi:10.1016/j.preteyeres.2017.10.006.
2. Jiang M-S, Xu X-L, Yang T, Li F, Zhang X-D. Comparison of choroidal thickness in preeclamptic, healthy pregnant, and nonpregnant women: a systematic review and meta-analysis. Ophthalmic Res. 2019; 62:1-10.
3. Dadaci Z, Alptekin H, Acir NO, Borazan M. Changes in choroidal thickness during pregnancy detected by enhanced depth imaging optical coherence tomography. Br J Ophthalmol. 2015; 99:1255-1259.
4. Nickla DL, Wallman J. The multifunctional choroid. Prog Retin Eye Res. 2010; 29:144-168.
5. Jendzjowsky NG, Steinback CD, Herman RJ, Tsai WH, Costello FE, Wilson RJA. Functional-optical coherence tomography: a non-invasive approach to assess the sympathetic nervous system and intrinsic vascular regulation. Front Physiol. 2019; 10:1146. doi: 10.3389/fphys.2019.01146

ARE CHANGES DESCRIBED IN THE MACULA FOUND ELSEWHERE IN THE EYE?

To answer this question, we returned to early study data where we measured both the macula and optic nerve head (ONH). Applying the observed test re-test coefficient of repeatability (COR) and the convention of 2 times the COR in >33% of measurements as used in the macular clinical decision support tool for these tissues (note, this has never been validated), findings would require ≥6 µm change in 2 of the four quadrants of the retinal nerve fiber layer (RNFL), ≥10 µm change in RNFL measurements in 2 of 4 sectors at the calculation circle and ≥120 µm change in 2 of 4 sectors of the neuro-retinal rim in order to be significant. Four participants in our HDP study had complete imaging data at the ONH (i.e., <20 gestation, 20-weeks to delivery, at delivery and a non-pregnant image) and there was partial data on 10 others. Of the 4 with complete data, 1 was a non-hypertensive control, 2 had de novo HDP and the third had HDP superimposed on chronic hypertension. The neuro-retinal rim was not included in these analyses because of excessive variability.

We have examined these data and observed isolated instances where the decision support tool signals possible thinning and/or thickening in 1 or more regions. However, only 1 of the 14 individuals had concordant thinning or thickening at both the macula and the ONH. In the vast majority of cases there was nothing to suggest the changes we report at the macula are reflected in the ONH. The thickness of the peripapillary RNFL is reported to be identical in normal pregnancy and pregnancy complicated by preeclampsia; but both are thicker than post pregnancy measurements[1] and measurements in non-pregnant normal controls[2]. Vascular perfusion density of the peripapillary capillary network increases in preeclampsia whereas vascular perfusion densities of the superficial and deep capillary plexuses decrease[3]. This may explain why there are

difference in response of these tissues in pregnancy and in HDP.

References for Are Changes Described In The Macula Found Elsewhere?

1. Arab M, Entezari M, Ghamary H, Ramezani A, Ashori A, Mowlazadeh A, et al. Peripapillary retinal nerve fiber layer thickness in preeclampsia and eclampsia. Int Ophthalmol. 2018; 38:2289-2294.
2. Atas M, Acmaz G, Aksoy H, Demircan S, Atas F, Gulhan A, et al. Evaluation of the macula, retinal nerve fiber layer and choroids in preeclampsia, healthy pregnant and healthy non-pregnant women using spectral-domain optical coherence tomography. Hypertens Pregnancy. 2014; 33:299-310.
3. Ciloglu E, Okcu NT, Dogan NÇ. Optical coherence tomography angiography findings in preeclampsia. Eye. 2019; 33:1946-1951.

WHAT TO TAKE AWAY FROM THIS STUDY

It must be emphasized that we are not directly measuring the autoregulatory reflex. To be clear, we are measuring the effects of autoregulation on a very unique end-tissue that possesses many of the important characteristics of brain, including the presence of high-resistance blood-tissue barriers, and confined within a closed space. Primates are the only species with a macula. Thus, to understand how the human brain autoregulates its microcirculation, you need to observe it in vivo. OCT of the macula provides that opportunity. We have shown that OCT is able to reliably measure small changes in the macula that accompany functional changes in autoregulation of the microcirculation of the central retinal artery of the eye. We have observed and described both physiologic gain of autoregulation and pathophysiologic breach of both upper and lower thresholds of autoregulation in a hypertensive emergency. These findings have internal validity on 2 independent analyses, but arise from very small subject numbers with high heterogeneity and need validation by others before they can be accepted. We hope this opens a door to better understanding and treatment of patients with hypertensive emergencies, including HDP.

Finally, transient loss of macular thinning was observed in 5 of 11 non-hypertensive controls and 2 of 16 women with HDP superimposed on chronic hypertension. When this occurred, it was observed in both eyes and was coincident with a 13-22 mmHg reduction in systemic blood pressure in 3 participants. Two participants experienced transient loss of thinning while severely iron deficient, which corrected with iron replacement. A definitive cause was not found in the 2 remaining participants. Importantly, loss of thinning returned to its usual thinned state by the time of delivery in all 7 cases. Thus, we attribute loss of thinning to loss-of-gain of auto-regulation in these individuals, as the response in all returned to thinning by the time of delivery.

These observations are important for a number of reasons. First, they point to the presence

of a highly regulated microvascular reflex whose effect extends broadly and evenly across all regions of the neurosensory retina. Secondly, loss of macular thinning alone is not sufficient to define capillary leak, nor the presence of retinal edema. Indeed, it may simply reflect loss-of-gain of vasoconstriction at lower hemodynamic load. On the other hand, loss of thinning coincident with or leading to a meaningful rise in systemic blood pressure is highly predictive of progression to HDP. Finally, since hemodynamic load on the maternal circulation is high in late pregnancy, return of the macula to a predominantly thinned state at delivery suggests the microcirculation is intact and functioning appropriately.
